# A pilot study to assess the effect of integrating an automated speech‐based cognitive assessment into a brain health platform on PCP visits

**DOI:** 10.1002/alz70856_107056

**Published:** 2026-01-08

**Authors:** Vivian Vasallo, Nicklas Linz, Simona Schäfer, Vera Maljkovic, Kay Bhothinard, Russ Paulsen

**Affiliations:** ^1^ UsAgainstAlzheimer's, Washington, DC, USA; ^2^ ki:elements GmbH, Saarbrücken, Germany; ^3^ ki:elements GmBH, Saarbrücken, Germany; ^4^ Eisai, Inc, Nutley, NJ, USA

## Abstract

**Background:**

Alzheimer's disease is underdiagnosed, and symptoms are regularly detected late in the disease progression, highlighting the need for efficient screening options. One promising digital detection tool is the speech biomarker for cognition (ki:e SB‐C), which effectively detects memory impairment linked to Alzheimer's (Tröger et al., 2022). The BrainGuide^®^ platform by UsAgainstAlzheimer's reaches tens of thousands of people annually who are interested in their brain health. Integrating ki:e SB‐C into this widely used brain health platform could promote screening, encourage at‐risk users to seek evaluation from a primary care physician (PCP) and allow evaluation of efficacy.

**Method:**

The ki:e SB‐C was integrated into the BrainGuide platform, where users could access information and complete the ki:e SB‐C remotely. After completing the assessment, users received a report with their score and based on normative data, an estimation of their cognitive risk (low, medium, or high). Users were also invited to rate the usability of the assessment and indicate whether they planned to consult a doctor. User behavior, usability ratings, and ki:e SB‐C scores from a 10‐week trial period were then analyzed.

**Result:**

Out of 416 users (28%) who began the SB‐C assessment on BrainGuide, 154 completed it. The mean SB‐C score was 0.37 ± 0.32 (range 0‐1). Risk scores were: 42% high, 32% medium, and 26% low. Fifteen users rated usability, with an average score of 8.8/10. Of the 50 users who answered whether they would consult a doctor about memory concerns, 31 agreed. The study is ongoing.

**Conclusion:**

Individuals concerned about their cognition who visit a brain health web platform are generally open to taking a speech‐based test. Nearly 75% of those who completed the test performed in a range for their age and education that indicates moderate or high risk of impairment. Early findings indicate that the assessment might also influence the willingness to make an appointment with a PCP.

